# 

**Published:** 1893-12-09

**Authors:** 


CONTENTS.

**An Anxious Succession** - - - - 145
**Around the Hospitals** - - - - 146
**On Modern Progress in Ophthalmic Medicine and Surgery.** By Robert Brudenell Carter, F.R.C.S. - 147
**Modern Aspects of Appendicitis. II.** - - - 148
**The Study of Man.** II.—First Studies in Anthropology - 149
**The Field of Science** - - - - 150
**Annotations.**—Lord Salisbury's Despondency—A Medical Lovers' Quarrel—The Death of Professor Tyndall - 151
**Notes and News** - - - - 152
**The Hospital Clinic**—
The Treatment of Acute Maniacal Delerium (*concluded*) - 153
The London Hospital—Treatment of Erysipelas - - 153
**Editor's Letter-Box.** Mattei Remedies and Cure for Drunkenness—The Medical Defence Union and the London and Counties Medical Protection Society (Limited) - - 155
**Salicylic Acid for Preserving** - - - - 156
**The Institutional Workshop**—
Hospitals of the World—VI. Military and Naval Hospitals- 157
Hospital Construction—Hospital Convalescent Home, Parkwood, Swanley, Kent (*concluded*) - - - 158
Hospital Festivals and Meetings—The London Hospital—The London Dental Hospital Students' Dinner—The Surgical Aid Society - - - - 159
**Scraps and Gleanings** - - - - 160
**The Book World of Medicine and Science** - - - vii

EXTRA SUPPLEMENT.—THE NURSING MIRROR.

**News from the Nursing World.**—Novel Competition for Nurses—Christmas Festivities—The Late Miss Allen—The Scarcity of Nurses—A Seasonable Plea—Half the Battle—A Basis of Self-Help—In Southern Spain—To Pass the Time Waiting for Work—Hard Labour—Short Items - - xci
**On the Nursing of Diseases of the Stomach. V.**—Dyspepsia - - - - xciii
**Notes from Melbourne** - - - - xciii
**Women's Work.**—Female Medical Aid for the Women of India. By Mrs. Scharlieb, M.D. IV.—Europeans and Natives - xciv
**Nursing in Switzerland.** By Dr. Charles Kraft - - xciv
**Causerie from New England.**—Boston, Massachusetts - xcv
**Nursing in the South Sea Islands** - - - xcvi
**Wants and Workers; Minor Appointments** - - xcvi
**Everybody's Opinion.**—Workhouse Infirmaries—Asylum *v.* Hospital Training—Irish Distressed Ladies' Fund - - xcvii
**Experiences of a Lecturer in a Rural District** - xcviii
**Medico-Psychological Association of Great Britain and Ireland** - - - - xcviii
**The Book World for Women and Nurses** - - - xcix

